# Unveiling the Silent Threat: Exploring Hypertension Prevalence and Risk Factors Among University Students in Syria

**DOI:** 10.3389/ijph.2025.1607939

**Published:** 2025-02-03

**Authors:** Shuaib Alahmad, Abudlla Samman, Rawaa Al Kayali

**Affiliations:** ^1^ Faculty of Pharmacy, Al-Wataniya Private University, Hama, Syria; ^2^ Faculty of Medicine, Al Kalamoon University, Deir Atiyah, Syria; ^3^ Faculty of Pharmacy, University of Aleppo, Aleppo, Syria

**Keywords:** elevated pressure, hypertension, risk factors, Syria, university students

## Abstract

**Objective:**

The prevalence of hypertension has increased worldwide over the last decades. No data are available on the prevalence and correlates of hypertension and elevated blood pressure among young adults in Syria. This study aimed to measure the prevalence of hypertension and elevated blood pressure among university students and to identify the associated sociodemographic characteristics, and behavioral risk factors.

**Methods:**

This study was designed as a cross-sectional investigation with 1,100 subjects randomly selected from the students of Aleppo and Al-Wataniya universities. Risk factors, and measurement data were collected using a questionnaire. Hypertension was categorized according to ACC/AHA guidelines.

**Results:**

Out of 1,100 undergraduate university students, men made up 70.2% of the total respondents. The age of the participants ranged from 18 to 30 years with a mean age of 21 (±1.82). The prevalence of elevated blood pressure and hypertension was 27.7% and 15.9% respectively. The main common risk factors for elevated blood pressure and hypertension were sex, age, smoking, stress and BMI. Family history was only associated with hypertension.

**Conclusion:**

The prevalence of hypertension among undergraduate students was higher than in other countries which calls for urgent policy actions targeting this age group for early prevention of hypertension.

## Introduction

Hypertension (HTN) is defined as persistently high arterial blood pressure that is continuously above normal limits for an extended period of time [1]. It is a significant health burden worldwide and a non-communicable condition contributing to substantial morbidity and mortality [2]. The WHO estimates that one in eight deaths worldwide is attributable to hypertension [3]. Often referred to as the “silent killer,” hypertension can present with no apparent symptoms until severe complications, particularly of the cardiovascular system, arise [4]. The global prevalence of hypertension is on the rise, with recent estimates suggesting that approximately one-third of adults, or 1.28 billion people, live with hypertension, two-thirds of whom are found in low- and middle-income countries [5]. This may be due to several factors, such as socio-economic challenges, limited knowledge and screening, inadequately managed risk factors, high-stress levels, and insufficient healthcare services [6].

In 2003, the Joint National Committee on Prevention, Detection, Evaluation, and Treatment of High Blood Pressure (JNC 7) introduced the term “prehypertension” (PHTN) to raise awareness about individuals whose blood pressure (BP) is nearing abnormal levels. The goal was to prompt these individuals to adopt healthier lifestyles and prevent the progression of established hypertension [7]. Individuals with PHTN have a higher risk of progressing to HTN and an overall increased risk of cardiovascular events and mortality [8]. In 2017, the ACC/AHA replaced the term “prehypertension” with the term “elevated blood pressure.” The European Society of Hypertension (ESH) and In 2018, The European Society of Hypertension (ESH) revised their guidelines to classify “elevated pressure” into the “high normal” blood pressure category (Table 1) [9, 10].

**TABLE 1:** Blood pressure classification according to the American College of Cardiology (ACC)/American Heart Association (AHA) 2017 and European Society of Cardiology (ESC)/European Society of Hypertension (ESH) 2018 guidelines [9, 10] (Syria, 2023).

Category	SBP		DBP
ACC/AHA 2017			
Normal	<120	and	<80
Elevated	120–129	and	<80
Stage 1 hypertension	130–139	or	80–89
Stage 2 hypertension	≥140	or	≥90
ESC/ESH 2024			
Optimal	<120	and	<80
Normal	120–129	and/or	80–84
High-normal	130–139	and/or	85–89
Grade I hypertension	140–159	and/or	90–99
Grade 2 hypertension	160–179	and/or	100–109
Grade 3 hypertension	≥180	and/or	≥110
Isolated systolic hypertension	≥140	and	<90

While hypertension has been considered to be a disease associated with older age for a long time, recent studies have shown that it is becoming increasingly prevalent even in younger populations, such as higher education students [11]. Such a trend emphasizes the need to explore specific risk factors that affect blood pressure in young people. Established studies identify tobacco use, alcohol consumption, poor diet, physical inactivity, elevated BMI, stress, and high cholesterol levels as modifiable risk factors. Non-modifiable factors include age, sex, family history, and ethnicity [6]. Cost-effective primary prevention measures aimed at controlling modifiable risks are the key to preventing the debilitating complications of HTN [6, 12].

No previous study has been conducted in Syria on the prevalence and risk factors of hypertension among university students. This research attempts to investigate the risk factors associated with elevated and high blood pressure among Syrian university students. Such a study will provide significant insights into lifestyle influences, stressors, and possible strategies that can help to overcome this silent health crisis.

## Methods

### Study Design

A cross-sectional study was conducted to study the prevalence of abnormal hypertension among university students of the University of Aleppo and Al-Wataniya Private University. The University of Aleppo is located in northern Syria in the city of Aleppo. It currently includes twenty-seven colleges, twelve technical institutes, seven open education programs, a central library, a television radio center, and six educational hospitals. Al-Wataniya private university is located in the city of Hama and it includes seven colleges.

### Participants and Sampling

The research included students aged 18–30 years, of both sexes, who did not have any chronic diseases that could potentially affect blood pressure (such as cardiovascular disease, kidney disease, diabetes, hormonal problems, lupus, and scleroderma) or health conditions requiring ongoing medical treatment that could affect blood pressure such as NSAIDs or antidepressants. All students who met the inclusion criteria and consented to the study were included in our study.

The sample size was calculated using the Sample Size Calculator at Calculator.net using a 5% significance level, 40% prevalence of high blood pressure (WHO) [13], a 5% margin of error and a design effect of 2. The total number of undergraduate students at Aleppo Government University and Al-Wataniya Private University is approximately 123000. The minimum required calculated sample size was 767; a total number of 1,100 were recruited to increase the statistical power. Final-year pharmacy students were properly trained on the tools and field methods to perform the data collection processes.

### Data Collection

Data collection was conducted for 3 months from January 2023 to June 2023, using a self-constructed face-to-face interview questionnaire: each student took 20–30 min to complete the tool and to perform physical measurements in a private manner in the presence of researchers. The interview questionnaire was structured into three sections (socio-demographic characteristics, lifestyle questions, and measurements). A structured interview questionnaire sheet was designed by the researchers to meet the aims of the study. It was written in simple Arabic language to suit the level of understanding of the students. The Socio- Demographic characteristics included age, sex, university, type of college, place of residence, wealth status and a family history of hypertension. The health behaviors comprised smoking status, consumption of caffeinated drinks, physical activity, number of hours slept, stress and nutritional lifestyle. Finally, the measurements included weight (kg), and height (m). BMI and Blood pressure.

BMI was categorized according to the World Health Organization criteria, where a BMI of <25 kg/m^2^ is considered normal, a BMI between 25 and 29 kg/m^2^ is considered overweight, and a BMI≥ 30 kg/m^2^ is considered obese.

Hypertension categories were classified according to the 2017 ACC/AHA hypertension guidelines [9]. Individuals were classified according to their highest systolic or diastolic BP category. BP was measured according to the American Heart Association guidelines using a mercury column sphygmomanometer and a cuff appropriate for the participant’s arm circumference. Categories were not based on BP readings at a single point in time but rather on an average of three readings taken on separate occasions.

A pilot study was conducted on 10% of the total study sample. The aim was to test the suitability of the questionnaire for use in terms of its clarity of sentences, estimated time to complete the questionnaire, analytical steps, and comprehensive student answers. This study showed that overall the questionnaire was appropriate, and participants in the pilot study were excluded from the main study sample.

### Ethical Considerations

Ethical approval was granted by the pharmacy ethics committee at the University of Aleppo (SAP-13/V.19.1.2023, and by the general research administration ethics committee at Al-Wataniya Private University (WAP, 13/II.2.25.1.2023). All participants signed an informed consent prior to each interview and the anonymity of participants was ensured.

### Statistical Analysis

The data obtained were analyzed using the Statistical Package for the Social Sciences (SPSS, version 18.0). Descriptive statistics included frequencies, percentages, means and standard deviations (SD) and the crude associations of each risk factor variable with hypertension were determined using the χ^2^ test. The odds ratio and 95% CIs were estimated using multinomial logistic regression, which showed a significant association with a *p*-value < 0.05.

## Results

### Description of Study Population Characteristics

A total of 1,100 students aged ≥18 years were enrolled for hypertension screening. The majority, 772 (70.2%) of the students were men and 328 (29.8%) were women, with a male-to-female sex ratio of 2.4:1 The mean age of the students was 21 (±1.82) years. Approximately half of the enrolled students were studying in medical colleges and one-third of them lived in hostels. Approximately one-quarter of the participants, 22.7% had a low family income and 36.5% had a family history of hypertension.

In terms of health behaviors, 86.5% of the students followed a healthy diet, 27.2% were physically active, 63.6% were sleeping 6–8 h per night, approximately half of the students had caffeinated drinks and one-third of them were smokers, 29.8% of whom were men. More than half of the students felt stressed (58.6%). Of them, 72.9% were men and 49.3% were in medical colleges.

For all participants, weight and height were measured. The mean BMI was 22.73 (±2.7). The majority (76.4%) had a normal BMI, while 18.2% were overweight. The prevalence of obesity was only 1.5%, and underweight was 3.9%. The socio-demographic, behavioral and health characteristics of participants overall and according to sex are presented in Table 2. The characteristics of the respondents differed among women and men. Women were more likely to be more obese and more likely to be stressed. On the other hand, men were more likely to smoke than women and they were more engaged in physical activities.

**TABLE 2:** Characteristics of participants in total and based on sex (Syria, 2023).

Characteristics	Total No. (%) (1,100)	Women No. (%) (772)	Men. No. (%) (328)	P value
University				
University of Aleppo	900 (81.8)	661 (85.6)	239 (72.9)	<0.001
Al-Wataniya Private University	200 (18.2)	111 (14.4)	89 (27)	
Age group				
18–21	385 (34.9)	224 (29)	161 (49.1	<0.001
22–25	683 (62.1)	532 (68.9)	151 (46)	
26–30	32 (2.9)	16 (2.1)	16 (4.9)	
College				
Medical	520 (47.3)	348 (45.1)	172 (52.4)	0.014
Engineering	407 (37)	307 (39.8)	100 (30.5)	
Other	173 (15.7)	117 (15.2)	56 (17.1)	
Place of residence				
With family	802 (72.9)	565 (73.2)	237 (72.3)	0.75
At a hostel	289 (27.1)	207 (26.8)	91 (27.7	
Family wealth status				
Poor	250 (22.7)	179 (23.2)	71 (21.6)	0.85
Middle class	644 (58.6)	449 (58.2)	195 (59.2)	
Rich	206 (18.7)	144 (18.7)	62 (19.9)	
BMI				
Underweight	43 (3.9)	18 (2.3)	25 (7.6)	0.001
Normal	840 (76.4)	590 (76.4)	251 (76.5)	
Overweight	200 (18.2)	153 (19.8)	47 (14.3)	
Obesity	16 (1.5)	12 (1.4)	4 (1.2)	
Smoking				
No	728 (66.2)	444 (57.5)	284 (86.6)	<0.001
Yes	372 (33.8)	328 (42.5)	44 (13.4)	
Consumption of caffeinated drinks (More than 3 cups a day)				
No	510 (46.4)	344 (44.6)	166 (50.6)	0.07
Yes	590 (53.6)	428 (55.4)	162 (49.2)	
Physical activity				
No	797 (72.5)	551 (71.4)	246 (75)	0.21
Yes	303 (27.5)	204 (26.4)	99 (30.2)	
Family history of hypertension				
No	698 (63.5)	473 (61.3)	225 (68.6)	0.012
Yes	402 (36.5)	299 (38.7)	103 (31.4)	
Stress				
No	455 (41.4)	302 (39.1)	153 (46.6)	0.02
Yes	645 (58.6)	470 (60.9)	175 (53.4)	
Healthy nutrition				
No	149 (13.5)	86 (11.1)	63 (19.2)	<0.001
Yes	951 (86.5)	686 (88.9)	265 (80.8)	
Number of hours slept				
Less than 6 h	292 (26.5)	199 (25.8)	93 (28.4)	0.6
6–8 h	700 (63.3)	495 (64.1)	205 (62.5)	
More than 8 h	108 (9.8)	78 (10.1)	30 (9.1)	

### Prevalence of Normal Blood Pressure, Elevated Blood Pressure and Hypertension

The mean diastolic blood pressure measurement was 77.8 (±6.82) mmHg whereas the mean systolic blood pressure measurement was 122.46 (±10.97) mmHg.

**FIGURE 1:**
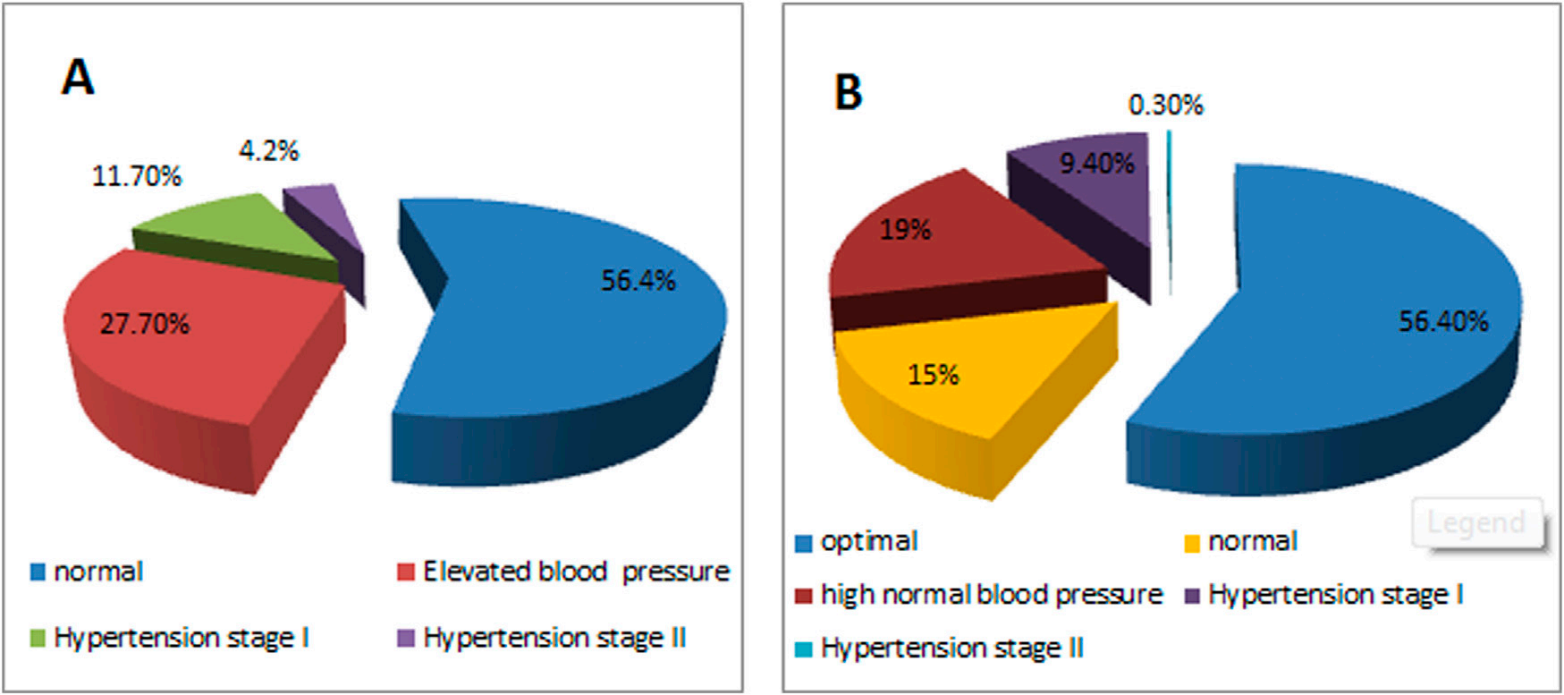
Distribution of blood pressure categories among study participants, **(A)** According to the American College of Cardiology (ACC)/American Heart Association (AHA) 2017 guidelines, **(B)** According to the European Society of Cardiology (ESC) 2018 guidelines (Syria, 2023).

The prevalence of ACC/AHA 2017 and ESC 2018 blood pressure categories among study participants is shown in Figure 1. The prevalence rate of normal blood pressure was similar (56.4%) in the two classifications. However, according to the ACC/AHA 2017 guidelines, the rates of elevated blood pressure and hypertension were 27.7% and 15.9% respectively. Meanwhile, according to the ESC 2018 guidelines, the rates of high normal blood pressure and hypertension were 18.80% and 9.7% respectively. Participants who had elevated blood pressure according to our measurement were advised to adopt a healthy lifestyle while those with hypertension were advised to undergo further clinical evaluation and treatment.

### Risk Factors Associated With Elevated Blood Pressure

**FIGURE 2:**
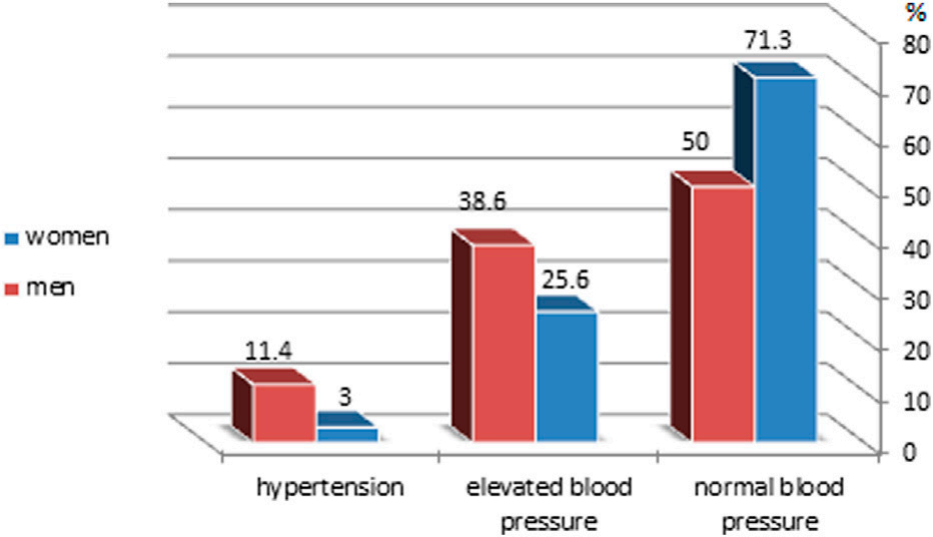
Distribution of blood pressure categories among study participants by sex according to the American College of Cardiology (ACC)/American Heart Association (AHA) 2017 guidelines (Syria, 2023).

The characteristics of all participants based on BP categories are presented in Table 3. The prevalence of elevated blood pressure and hypertension was similar in the following groups: type of college, place of residence, family wealth status, kind of nutrition, and number of hours slept. The prevalence of elevated blood pressure and hypertension was higher in men than women (Figure 2), older students (22–25 years old), smokers, stressed individuals, and obese individuals. Students with a family history of hypertension also exhibited higher rates of elevated blood pressure and hypertension.

**TABLE 3:** Characteristics of participants according to hypertension status (Syria, 2023).

Variable	No. (%)	Normal blood pressure No. (%)	Elevated blood pressure No. (%)	Hypertension No. (%)	Chi-square value P value
University					
University of Aleppo	900 (81.8)	504 (56)	243 (27)	153 (17)	7.3 0.3
Al-Wataniya Private University	200 (18.2)	121 (60.5)	57 (28.5)	22 (11)	
Sex					
Female subjects	328 (29.8)	234 (71.3)	69 (21)	25 (7.6)	47.8** <0.001
Male subjects	772 (70.2)	391 (50.6)	231 (29.9)	150 (19.4)	
Age group					
18–21	384 (34.9)	257 (66.8)	95(24.7)	32 (8.6)	38. 2** <0.001
22–25	683 (62.1)	346 (50.7)	201 (29.4)	136 (19.9)	
26–30	32 (2.9)	22 (68.8)	4 (12.5)	6 (12.5)	
College					
Medical	520 (47.3)	291 (56)	157 (30.2)	72 (11.9)	5.7 0.21
Engineering	407 (37)	299 (56.3)	104 (25.6)	74 (18.2)	
Other	137 (15.7)	105 (60.7)	39 (22.5)	29 (16.8)	
Place of residence					
With family	802 (72.9)	462 (57.6)	219 (27.3)	141 (15.1)	0.47 0.7
At a hostel	289 (27.1)	163 (54.7)	81 (27.2)	54 (18.1)	
Family wealth status					
Poor	250 (22.7)	137 (54.8)	67 (26.8)	46 (18.4)	3.6 0.47
Middle-class	644 (58.5)	370 (57.5)	176 (27.3)	98 (15.2)	
Rich	201 (18.7)	118 (57.3)	57 (27.7)	31 (15.0)	
BMI					
Underweight	43 (3.9)	30 (69.8)	6 (14)	7 (16.3)	23.4* <0.001
Normal	840 (76.4)	499 (59.3)	230 (27.3)	112 (13.3)	
overweight	200 (18.2)	91 (45.5)	59 (29.5)	50 (25.0)	
Obesity	16 (1.5)	5 (31.3)	5 (31.3)	6 (27.6)	
Smoking					
No	728 (66.2)	464 (63.7)	180 (24.7)	84 (11.6)	57.5** <0.001
Yes	372 (33.8)	161 (43.3)	120 (32.3)	91 (24.4)	
Consumption of caffeinated drinks (More than 3 cups a day)					
No	510 (46.4)	335 (56.7)	115 (22.5)	60 (11.7)	26.9 <0.08
Yes	590 (53.6)	290 (49.2)	185 (31.4)	35 ( 9.5)	
Physical activity					
No	797 (72.5)	440 (55.2)	216 (27.1)	141 (17.7)	9.5 0.4
Yes	303 (27.5)	185 (61.1)	84 (27.7)	34 (11.3)	
Family history of hypertension					
No	698 (63.5)	419 (60)	196 (28.1)	83 (11.9)	31.5 <0.001*
Yes	402 (36.5)	206 (51.2)	104 (25.9)	92 (22.9)	
Stress					
No	455 (41.4)	322 (70.8)	99 (21.8)	34 (7.5)	72.7 <0.001*
Yes	645 (58.6)	303 (47)	201 (31.2)	141 (21.9)	
Healthy nutrition					
No	149 (13.5)	95 (63.8)	37 (24.8)	17 (11.4)	6.5 0.2
Yes	951 (86.5)	530 (55.7)	263 (27.7)	158 (16.6)	
Number of hours slept					
Less than 6 hours	292 (26.5)	162 (55.6)	104 (35.6)	26 (8.9)	1.9 0.7
6–8 hours	700 (63.3)	392 (56%)	247 (35.3)	61(8.7)	
More than 8 hours	108 (9.8)	66 (61.1)	31 (28.7)	11 (10.2)	

Chi-square tests indicated that sex, age, family history of hypertension, smoking, stress, and BMI were significantly associated with elevated blood pressure (p < 0.001). Factors such as university, type of college, place of residence, family wealth status, nutrition habits, physical activity, and number of hours slept did not show statistically significant associations.

The sex-based analysis revealed different risk profiles. In women, BMI was significantly linked to hypertension, while smoking had no association. In men, smoking was significantly linked to hypertension, in contrast to women (Table 4).

**TABLE 4:** Distribution of hypertension categories according to sex between variable groups (Syria, 2023).

Variable	Female subjects				Male subjects			
	Normal blood pressure NO. (%)	Elevated blood pressure NO. (%)	Hypertension NO. (%)	χ2 value P value	Normal blood pressure NO. (%)	Elevated blood pressure NO. (%)	Hypertension NO. (%)	χ2 value P value
University								
University of Aleppo	304 (46)	207 (40.8)	87 (13.2)	33.3 0.7	195 (81.6)	41 (17.2)	3 (1.3)	46.8 0.2
Al-Wataniya Private University	82 (73.9)	28 (25.2)	1 (0.9)		39 (43.8)	43 (48.3)	7 (7.9)	
Age group								
18–21	136 (60.7)	78 (34.8)	10 (4.5)	25.7 <0.001*	118 (73.3)	39 (24.2)	4 (2.5)	7.9 0.05*
22–25	241 (45.3)	217 (40.8)	74 (13.9)		103 (68.2)	43 (28.5)	5 (3.3)	
26–30	9 (56.3)	3 (18.8)	4 (25)		13 (81.3)	2 (12.5)	1 (6.3)	
College								
Medical	177 (50.9)	139 (39.9)	32 (9.2)	8.1 0.09	110 (64)	54 (31.4)	8 (4.7)	7.4 0.15
Engineering	147 (47.9)	124 (40.4)	36 (11.7)		82 (82)	16 (16)	2 (2)	
Other	62 (53)	35 (29.9)	20 (17.1)		42 (75)	14(25)	0 (0)	
Place of residence								
With family	286 (50.6)	216 (38.2)	63 (11.2)	0.35 0.8	171 (72.2)	59 (24.9)	7 (3)	0.27 0.9
At a hostel	100 (48.3)	82 (39.6)	25 (12.1)		63 (69.)	225 (27.5)	3 (3.3)	
Family wealth status								
Poor	88 (49.2)	69 (38.5)	22 (12.3)	5.5 0.24	48 (67.6)	21 (29.6)	2 (2.8)	2.1 0.75
Middle-class	225 (50.1)	181 (40.3)	43 (9.6)		143 (73.3)	45 (23.1)	7 (3.6)	
Rich	73 (50.7)	48 (33.3)	23 (16)		43 (69.4)	18 (29)	1 (1.6)	
BMI								
Underweight	12 (66.7)	5 (27.8)	1 (5.6)	17.2 0.009*	18 (72)	6 (24)	1 (4)	5.1 0.5
Normal	310 (52.5)	223 (37.8)	57 (9.7)		184 (73.3)	60 (23.9)	7 (2.8)	
overweight	61 (39.9)	65 (42.5)	27 (17.6)		30 (63.8)	15 (31.9)	2 (4.3)	
Obesity	3 (27.3)	5 (45.6)	3 (27.3)		2 (40)	3 (60)	0 (0)	
Smoking								
No	247 (55.6)	165 (37.2)	32 (7.2)	19.8 <0.09	215 (75.7)	62 (21.8)	7 (2.5)	23.2 <0.001*
Yes	139 (42.4)	133 (40.5)	56 (17.1)		19 (43.2)	22 (50)	3 (6.8)	
Consumption of caffeinated drinks (More than 3 cups a day)								
No	187 (54.4)	127 (36.3)	30 (8.7)	0.7 0.07	142 (85.5)	22 (13.3)	2 (1.2)	33.3 0.06
Yes	199 (46.5)	171 (40)	58 (13.6)		92 (56.8)	62 (38.3)	8 (4.9)	
Physical activity								
No	134 (60.6)	70 (31.7)	17 (7.7)	14.6 0.05	50 (61)	28 (34.1)	4 (4.9)	5.9 <0.01*
Yes	252 (45.7)	228 (41.4)	71 (12.9)		184 (74.8)	56 (22.8)	6 (2.4)	
Family history of hypertension								
No	249 (52.6)	190 (40.2)	34 (7.2)	21.5 <0.001*	67 (65)	29 (28.2)	7 (6.8)	8.1 0.017*
Yes	137 (45.8)	108 (36.1)	54 (18.1)		167 (74.2)	55 (24.4)	3 (1.3)	
Stress								
No	201 (66.6)	90 (29.8)	11 (3.6)	63.3 <0.001*	116 (66.3)	50 (28.6)	7 (6.8)	8.1 0.018*
Yes	185 (39.4)	208 (44.3)	77 (16.4)		118 (77.1)	34 (22.2)	1 (0.7)	
Healthy nutrition								
No	51 (59.3)	31 (36)	4 (4.7)	5.7 0.06	190 (71.7)	67 (25.3)	8 (3)	0.08 0.95
Yes	335 (48.8)	267(38.9)	84 (12.2)		44 (69.8)	17 (27)	2 (3.2)	
Number of hours slept								
Less than 6 hours	100 (0.3)	78 (39.2)	21 (10.6)	3.25 0.5	62 (66.7)	26 (28)	5 (5.4)	5.1 0.5
6–8 hours	241 (48.7)	191 (39.8)	57 (11.5)		151 (73.7)	50 (24.4)	4 (2)	
More than 8 hours	45 (57.7 )	23 (29.5)	10 (12.8)		21 (70)	8 (26.7)	1 (3.3)	

### Multivariate Logistic Regression Analysis

All variables with significant chi-square associations were included in a multivariate logistic regression (Table 5). Significant predictors of both elevated blood pressure and hypertension included sex, age, smoking, BMI, and stress. A family history of hypertension was a significant predictor only for hypertension. Male participants had higher odds of both elevated blood pressure (OR: 1.8, 95% CI: 1.32–2.69, p < 0.001) and hypertension (OR: 3.14, 95% CI: 1.48–6.67, p = 0.003). Individuals aged 26–30 years had approximately 1.5 times higher odds of hypertension (OR: 1.32, 95% CI: 0.08–1.04, p = 0.03). Obesity significantly increased the odds of both elevated blood pressure (OR: 3.99, 95% CI: 1.02–15.3, p = 0.05) and hypertension (OR: 9.24, 95% CI: 1.08–78.9, p = 0.02). Stressed participants were twice as likely to have elevated blood pressure (OR: 2.09, 95% CI: 1.58–2.77, p < 0.001) and nearly seven times more likely to have hypertension (OR: 6.6, 95% CI: 3.36–12.97, p < 0.001). Smokers had increased odds of both elevated blood pressure (OR: 1.46, 95% CI: 1.08–1.98, p < 0.01) and hypertension (OR: 2.3, 95% CI: 1.33–3.9, p < 0.02).

**TABLE 5:** Logistic regression analysis of total participants for correlation of elevated blood pressure compared to normal blood pressure and hypertension compared to normal blood pressure (Syria, 2023).

Characteristics	Elevated blood pressure	*P* value	Hypertension	*P* value
	OR (95% CI)		OR (95% CI)	
University				
Al-Wataniya Private University	Reference group		Reference group	
University of Aleppo	1.27 (0.88–1.8)	0.18	1.14 (0.48–2.7)	0.96
Sex				
Female subjects	Reference group		Reference group	
Male subjects	1.8 (1.32–2.69)	<0.001*	3.14 (1.48–6.67)	0.003*
Age group				
18–21	Reference group		Reference group	
22–25	3.05 (0.11–8.4)	0.11	0.83 (0.27–2.59)	0.75
26–30	2.28 (0.82–6.34)	0.06	1.3 (0.08–1.04)	0.03*
Family history of hypertension				
No	Reference group		Reference group	
Yes	1.01 (0.76–1.35)	0.9	2.7 (1.66–4.34)	<0.001*
Smoking				
No	Reference group		Reference group	
Yes	1.46 (1.08–1.98)	0.02*	2.29 (1.35–3.88)	0.02*
Consumption of caffeinated drinks (More than 3 cups a day)				
No	Reference group		Reference group	
Yes	1.42 (1.07–1.98)	0.015*	1.34 (0.85–2.41)	0.17
Physical activity				
Yes	Reference group		Reference group	
No	1.24 (0.90–1.70)	0.12	1.21 (0.69–2.22)	0.51
Stress				
No	Reference group		Reference group	
Yes	2.09 (1.58–2.77)	<0.001*	6.6 (3.36–12.97)	<0.001*
Healthy nutrition				
Yes	Reference group		Reference group	
No	1.04 (0.67–1.56)	0.84	1.5 (0.63–4.02)	0.25
BMI				
Underweight	Reference group		Reference group	
Normal	1.13 (0.52–2.40)	0.74	0.80 (0.17–3.90)	0.78
Overweight	1.76 (0.79–3.88)	0.16	1.88 (0.38–9.46)	0.43
Obesity	3.99 (1.02–15.3)	0.05*	9.24 (1.08–78.9)	0.02*

### Sex-Based Differences and Determinants of Hypertension

Multivariate regression results for hypertension based on sex are shown in Table 6. Family history of hypertension and stress were common predictors for both sexes. In men, smokers and those not engaging in physical activities were at higher risk for hypertension. For women, obesity and stress were significantly associated with a higher risk of hypertension.

**TABLE 6:** Logistic regression analysis according to sex for correlates of elevated blood pressure compared to normal blood pressure (Syria, 2023).

Characteristics	Female subjects	*P* value	Male subjects	*P* value
	OR (95% CI)		OR (95% CI)	
University				
Al-Wataniya Private University	Reference group		Reference group	
University of Aleppo	2.09 (1.22–3.56)	0.7	0.29 (0.153–0.56)	0.2
Age group				
18–21	Reference group		Reference group	
22–25	2.97 (0.76–11.58)	0.11	1.80 0.36- (9.29)	0.49
26–30	2.33 (0.58–9.26)	0.23	1.87 0.36- (9.68)	0.45
Family history of hypertension				
No	Reference group		Reference group	
Yes	1.02 (0.73–1.43)	0.8	1.29 (0.71–2.36)	0.8
Smoking				
No	Reference group		Reference group	
Yes	1.03 (0.72–1.48)	0.9	1.74 (0.78–3.85)	0.05*
Consumption of caffeinated drinks (More than 3 cups a day)				
No	Reference group		Reference group	
Yes	0.85 (0.6–1.19)	0.35	0.3 (0.177–0.60)	0.1
Physical activity				
Yes	Reference group		Reference group	
No	1.21 (0.63–2.32)	0.5	1.6 (1.17–2.44)	0.008*
Stress				
No	Reference group		Reference group	
Yes	2.3 (1.66–3.22)	<0.001*	1.42 (0.78–2.66)	0.2
Healthy nutrition				
Yes	Reference group		Reference group	
No	0.98 (0.59–1.6)	0.9	0.96 (0.46–1.97)	0.9
BMI				
Underweight	Reference group		Reference group	
Normal	1.15 (0.37–3.59)	0.70	0.94 (0.28–2.50)	0.76
Overweight	1.6 (0.49–5.21)	0.43	0.91 (0.26–3.28))	0.89
Obesity	4.7 (0.76–30.37)	0.09	3.46 (0.34–35.26)	0.2

## Discussion

To the best of our knowledge, this is the first study to investigate the prevalence of elevated blood pressure and hypertension among young adults in Syria. According to the WHO, the prevalence of hypertension among Syrians aged 30–79 years is approximately 40% [13]. Our study reveals that the prevalence of elevated blood pressure and hypertension among young adults is not as low as generally expected, with overall prevalence rates of 27.7% and 15.9%, respectively according to the ACC/AHA 2017 guidelines [9]. These guidelines are more stringent than the ESH/ESC classification, resulting in higher prevalence rates (Figure 1). Muntner et al. compared U.S. and European guidelines, and they reported an increase in hypertension prevalence from 32% as per the ESC guidelines to 46% as per the ACC/AHA guidelines in the U.S. community [14]. So, the U.S. guidelines classify a large portion of the population as hypertensive potentially leading to earlier intervention and use of anti-hypertension medication to prevent the adverse complications of high blood pressure on the cardiovascular system and other organs [9]. In contrast, the European guidelines aim to prevent overdiagnosis and overtreatment by maintaining higher thresholds and prioritizing lifestyle changes unless the patient has additional cardiovascular risk factors [10].

According to the ESC/ESH guidelines, the prevalence of normal high blood pressure among our university students (18.8%) is comparable to reported figures in other countries, such as Saudi Arabia (17%) and Iraq (17.2%) but is higher than China (4.3%) (15–17). Similarly, the prevalence of hypertension in our study (9.7%) exceeds that reported in Vietnam (1.4%), and is lower than in Ethiopia (17.9%) and Bahrain (30.7%) [18–20].

These differences in prevalence may be attributed to differences in research methodologies, sampling methods, time frames, cultural and social factors, and divergent cut-off values for defining hypertension.

The determinants of elevated blood pressure and hypertension observed in our study were mostly identical. Significant predictors included sex, age, smoking status, BMI, and stress, aligning with findings from previous research [16–20]. Consistent with earlier studies, the prevalence of elevated blood pressure and hypertension was higher in men compared to women (38.6, 25.6) (11.4, 3) [15–21]. Blood pressure is influenced by sex, and this variation is likely due to hormonal differences such as the protective vasodilatory effect of estrogen in premenopausal women, [22]. Additionally, men in our study had higher rates of smoking (42.5%) compared to women (13.4%), which is a well-known risk factor for hypertension [17, 18, 21].

Smoking and elevated nicotine levels have an acute hypertensive effect due to increased adrenaline levels and are also associated with vascular endothelial dysfunction and inflammation [23, 24]. Conversely, in a British study conducted by Premasta et al., no statistically significant differences were recorded in blood pressure values between smokers and non-smokers [25]. In addition, a Chinese study reported a negative association between smoking and blood pressure, with current smokers having lower blood pressure than non-smokers and former smokers; smoking cessation was found to be significantly associated with an increased risk of high blood pressure [26].

Overweight and obesity have been previously identified as risk factors for both elevated blood pressure and hypertension [16, 18, 19]. The mechanisms by which obesity causes hypertension include alterations in adipose-derived hormones, insulin resistance, sympathetic nervous system overactivation, stimulation of the renin-angiotensin-aldosterone system, and renal sodium retention [27].

Consistent with several previous studies, a family history of hypertension was a significant determinant of hypertension in our study [20, 22, 24]. Hypertension tends to run in families due to shared genes, habits, environment, and lifestyle, predisposing individuals to hypertension and related conditions [28].

Chronic stress was positively associated with the development of hypertension, as found in a few previous studies [16, 18, 20, 29]. In Syria, prolonged exposure to psychological stress due to the armed conflict over the past decade may have contributed to the development of hypertension in young people [34, 35], paralleling findings from Ethiopia [30]. Chronic stress can alter cortisol levels and increase sympathetic nervous system activity, leading to high blood pressure [31, 32].

Interestingly, our study did not find significant differences in the prevalence of hypertension between students from public and private universities, or between medical and non-medical students, contrary to previous studies [19].

Several studies indicated that unhealthy lifestyles, characterized by physical inactivity, unhealthy diets and smoking, all of which are risk factors for hypertension, are prevalent among young people in developing countries [6, 33]. Our study showed the importance of physical activity as a risk factor for hypertension in both women and men.

In contrast to previous studies, nutrition habits, consumption of caffeinated drinks, and family wealth status were not defined as risk factors for elevated blood pressure or hypertension [20, 21]. However, other studies reported the same results [16, 18, 19]. Similarly, the number of hours slept was not associated with hypertension, although sleep duration was reported to be associated with high blood pressure [34], which may be due to the fact that Two-thirds of the participants slept (6–8) hours.

The sex-based analysis showed differences between women and men in terms of hypertension risk factors due to hormonal differences. The same results were reported in a previous study from Indonesia [35].

A cross-sectional survey in Syria highlighted the good knowledge and awareness of hypertension among adults aged 18 years and above. The study found that university-educated participants had higher knowledge of hypertension with an overall knowledge score of 89%. Even so, the participants still engaged in behaviors that increased the risk of hypertension, as 18.6% of the participants were smokers, with a significant proportion adding salt to their meals and consuming low amounts of fruits contributing to the risk of developing the condition [36]. These findings along with ours emphasize the urgent need to strengthen public health initiatives to promote the early detection and prevention of hypertension, among young adults. Moreover, the study underscores the importance of further research to identify risk factors and inform nationwide intervention strategies.

### Study Strengths and Limitations

The strength of our study lies in its extensive sample size in that it gathered data on both men and women from a public university and a private university, encompassing different socioeconomic statuses. However, the cross-sectional design used only describes the prevalence and correlation of risk factors related to elevated blood pressure and hypertension, not causality. In addition, the effects of sodium intake, cholesterol and triglyceride levels on elevated blood pressure and hypertension were not studied in our research due to limited resources. Furthermore, our sample may not be representative of all Syrian youth and further studies should be conducted to support our results. Despite these limitations, this study emphasizes the critical need for early screening for elevated blood pressure and hypertension and to reduce the burden of hypertension in the younger population.

### Conclusion

In summary, the prevalence of elevated blood pressure and hypertension among undergraduate students was higher than in some other countries according to U.S. guidelines. The prevalence of hypertension was much lower according to the ESH 2018 guidelines. Sex, age, smoking status, physical activity, stress and BMI were the determinants of hypertension for all participants. Sex-based risk factors revealed differences between men and women. The findings of the present study thus call for urgent sex-based policy actions targeting this age group for early prevention, detection, and treatment of hypertension. Addressing the “silent killer” should be a priority for healthcare providers to prevent and control further serious clinical complications among young people. Prevention and control through collaboration between various sectors such as parents and universities are needed to promote healthy lifestyles for combating this disease. Further expanded studies should be conducted in Syria to better understand how to identify risk factors for this disease so that early intervention can be conducted nationwide.

## Data Availability

Data will be made available on request.
